# Analysis of Novel *NEFL* mRNA Targeting microRNAs in Amyotrophic Lateral Sclerosis

**DOI:** 10.1371/journal.pone.0085653

**Published:** 2014-01-15

**Authors:** Muhammad Ishtiaq, Danae Campos-Melo, Kathryn Volkening, Michael J. Strong

**Affiliations:** 1 Molecular Medicine Research Group, Robarts Research Institute, Western University, London, Ontario, Canada; 2 Department of Clinical Neurological Sciences, Schulich School of Medicine and Dentistry, Western University, London, Ontario, Canada; Hertie Institute for Clinical Brain Research and German Center for Neurodegenerative Diseases, Germany

## Abstract

Amyotrophic lateral sclerosis (ALS) is a fatal disease characterized by progressive motor neuron degeneration and neurofilament aggregate formation. Spinal motor neurons in ALS also show a selective suppression in the levels of low molecular weight neurofilament (*NEFL*) mRNA. We have been interested in investigating the role of microRNAs (miRNAs) in *NEFL* transcript stability. MiRNAs are small, 20–25 nucleotide, non-coding RNAs that act as post-transcriptional gene regulators by targeting the 3′ untranslated region (3′UTR) of mRNA resulting in mRNA decay or translational silencing. In this study, we characterized putative novel miRNAs from a small RNA library derived from control and sporadic ALS (sALS) spinal cords. We detected 80 putative novel miRNAs, 24 of which have miRNA response elements (MREs) within the *NEFL* mRNA 3′UTR. From this group, we determined by real-time PCR that 10 miRNAs were differentially expressed in sALS compared to controls. Functional analysis by reporter gene assay and relative quantitative RT-PCR showed that two novel miRNAs, miR-b1336 and miR-b2403, were downregulated in ALS spinal cord and that both stabilize *NEFL* mRNA. We confirmed the direct effect of these latter miRNAs using anit-miR-b1336 and anti-miR-b2403. These results demonstrate that the expression of two miRNAs (miRNAs miR-b1336 and miR-b2403) whose effect is to stabilize *NEFL* mRNA are down regulated in ALS, the net effect of which is predicted to contribute directly to the loss of *NEFL* steady state mRNA which is pathognomic of spinal motor neurons in ALS.

## Introduction

Amyotrophic lateral sclerosis (ALS; Lou Gehrig's disease) is an adult onset neurodegenerative disease characterized by progressive muscle weakness which leads to paralysis and ultimately death by respiratory failure [1], [2]. ALS is a complex multifactorial disease for which the molecular basis remains elusive. Although most cases are diagnosed as sporadic (sALS), with only 10% cases traditionally being thought to be familial (fALS), there is an increasing awareness of genetic alterations underpinning a greater number of clinically sporadic cases [3].

Neuronal cytoplasmic inclusions (NCIs) in motor neurons are a neuropathological hallmark of ALS, including aggregates containing neurofilament (NF) [4] or RNA binding proteins such as TAR DNA binding protein of 43 kDa (TDP-43) [5], [6], fused in sarcoma/translocated in liposarcoma (FUS/TLS) [7], [8] and Rho guanine nucleotide exchange factor (RGNEF) [9]. In addition, motor neurons show a selective decrease in the steady state of low molecular weight NF (*NEFL*) mRNA levels thus altering the stoichiometry of NF expression [10], [11], [12], [13]. This is consistent with the hypothesis that alterations in RNA metabolism play a central role in ALS [14]. This hypothesis is further supported by the observation that the stability of *NEFL* mRNA is regulated by several RNA binding proteins that have been linked to the pathogenesis of ALS, including mutant superoxide dismutase 1 (mtSOD1), TDP-43 and RGNEF [9], [15], [16], [17]. Both mtSOD1 and RGNEF destabilize *NEFL* mRNA, while TDP-43 stabilizes the transcript [9], [16], [18].

We have previously shown that RNAse pre-treatment of ALS-derived spinal cord lysates restores *NEFL* mRNA stability, suggesting that an RNA species may also play a role in the determination of *NEFL* mRNA stability [19]. We have also shown a preferential colocalization between XRN1 and *NEFL* mRNA in spinal motor neurons in ALS, suggesting that *NEFL* is preferentially sequestered to processing bodies and thus targeted for degradation [19]. Based on these findings, we have been investigating the role of microRNAs (miRNAs) in the stability of *NEFL* mRNA.

MiRNAs are small non-coding RNAs originally discovered in *Caenorhabditis elegans.* They are highly conserved, strong post-transcriptional gene expression regulators found in organisms from algae to humans [20], [21], [22], [23], [24], [25]. These miRNAs can stabilize or destabilize the transcript or enhance translational activity by targeting mRNA at either the 3′or 5′UTR [26], [27], [28]. MiRNAs play a key role in neuronal cell identity [29] and alterations in miRNA function have been reported in a number of neurodegenerative diseases [30].

In this study, we identified novel miRNAs from a small RNA library of spinal cord tissue. Among 80 putative novel miRNAs, we obtained a panel that have miRNA response elements (MREs) within the *NEFL* mRNA 3′UTR and which are differentially expressed in ALS compared to controls. From this group, we performed functional analyses and found that two novel miRNAs, miR-b1336 and miR-b2403, were able to modulate *NEFL* mRNA stability in a manner consistent with the selective suppression of *NEFL* mRNA in ALS spinal motor neurons.

## Materials and Methods

### Tissues

We used ventral lumbar spinal cord samples from sALS patients and age-matched, neurologically intact control individuals for the small RNA library and real-time PCR (Table 1). All ALS cases were both clinically and neuropathologically confirmed using the El Escorial Criteria (World Federation of Neurology Research Group on Neuromuscular Disease, 1994). All research was approved by “The University of Western Ontario Research Ethics Board for Health Sciences Research Involving Human Subjects (HSREB)”. Written consent for autopsy was obtained from the next of kin at the time of death or from the patient antemortem in accordance with the London Health Sciences Centre consent for autopsy. Cases were genotyped and confirmed to have no known mutations in *SOD1*, *TARDBP*, *FUS/TLS*, or expanded repeats in *C9orf72* (Dr. Rosa Rademakers, Mayo Clinic, Jacksonville, Florida).

**Table 1:** Patient demographics of samples used in construct the small RNA libraries.

Case	Gender	Age of death (yrs)	Site of onset	Duration (yrs)
Control	F	62	—	—
Control	F	53	—	—
Control	M	61	—	—
ALS	M	67	Upper limb	3
ALS	F	49	Bulbar	2
ALS	M	50	Bulbar	1

### MiRNA isolation

Small RNAs were isolated for the library and real-time PCR using the MirVana miRNA isolation kit (Life Technologies Inc., Ambion, Burlington, ON, Canada) following manufacturer's instructions. Briefly, spinal cord tissue stored at −80°C was placed in 10 volumes of lysis/binding buffer on ice and homogenized. After organic extraction with acid-phenol:chloroform, miRNA isolation was performed by precipitating with ethanol and purifying over glass-fiber filters. Yield and purity of the miRNA enriched fraction was measured by UV absorbance at OD260/280.

### Small RNA library and identification of miRNAs

Sequencing libraries were generated by The Hospital for Sick Children (Toronto, ON, Canada) using a modification of the SOLiD™ Small Expression Kit (SREK) (Life Technologies Inc., Applied Biosystems, Burlington, ON, Canada). Briefly, MirVana-enriched miRNA fractions from spinal cord were hybridized and ligated to the adaptor mix. Next, the small RNA population was reverse transcribed and the cDNA library was amplified. After size selection of the amplified cDNA library by PAGE, libraries were quantified and samples were sequenced.

MiRNA identification and annotation was performed by InteRNA Technologies BV (Utrecht, The Netherlands) (see figure S1 for library details [31], [32]). Novel sequences were confirmed for the presence of miRNA structures by using Computational Identification of microRNA (CID, Center for Computational Biology and Bioinformatics) [33] and mireval (http://tagc.univ-mrs.fr/mireval) [34]. The Mfold web server (http://mfold.rit.albany.edu/?q=mfold/RNA-Folding-Form) was used to obtain for RNA secondary structures [35].

### MiRNA Target Analysis

*NEFL* mRNA 3′UTR was examined manually in the prediction of miRNA recognition elements according to miRanda and Targetscan algorithms [36], [37],[38]. Eighty putative novel miRNAs were analyzed considering the criterion of the seed sequence. We allowed only seed sequences of 7 nucleotides or more in our predictions, beginning at either position +1, +2 or +3 relative to the miRNA 5′-end [37], [39].

### Real-Time PCR

MiRNA-enriched samples were reverse transcribed using the miRNA reverse transcription kit according to manufacturer's protocols (Life Technologies Inc., Applied Biosystems). Preamplification of miRNA cDNAs was performed by TaqMan Preamp master mix kit, and real-time PCR performed with the TaqMan Fast Universal PCR Master Mix (2X) No AmpErase UNG (Roche, Branchburg, New Jersey) using a 7900 HT Real Time PCR system. Custom designed Taqman miRNA assays were used for detection of selected novel miRNAs in real-time PCR (Life Technologies Inc., Applied Biosystems). RNU6 was used as endogenous control.

Analysis of the relative expression data was performed using the 2^−ΔΔCT^ method. The fold change was calculated as log_10_ RQ where RQ is 2 ^−ΔΔCT^. Log_10_RQ correlates directly with up- (positive value) and down-regulation (negative value). A value of 1 in log_10_RQ means a 10 fold increase in expression. Similarly, a value of -1 represents 10 fold less expression compared to control. A miRNA was considered differentially expressed if the expression level was significantly different in at least 3 out of 5 sALS spinal cords.

### Plasmid construction

Reporter plasmid was constructed by inserting human *NEFL* mRNA 3′UTR (1838 bases; GenBank NM_006158) between NheI and SalI sites downstream of the firefly luciferase gene in the pmirGLO vector (Promega, Madison, WI, USA) pmirGLO-*NEFL* 3′UTR).

### Luciferase reporter assay

HEK293T cells were seeded in a 96-well plate at a density of 9×10^3^ cells/well 24 hours before transfection. Cells were co-transfected with 100 nM of wild type or mutant pre-miRNAs (Life Technologies Inc., Ambion, Burlington, ON, Canada) and 3.47 fmol of pmirGLO-*NEFL* 3′UTR using Lipofectamine 2000 reagent (Life Technologies Inc., Invitrogen, Burlington, ON, Canada) according to the manufacturer's instructions.

Luciferase activity was measured 24 hours post transfection in a luminometer (Turner Biosystem inc.) using Dual-Glo Luciferase Assay System (Promega, Turner Biosystems, Madison, WI, USA). Firefly luciferase activity was normalized to Renilla luciferase activity. Data were also normalized to the impact of each miRNA on the luciferase mRNA without *NEFL* 3′UTR to obtain the specific effect on the *NEFL* mRNA 3′UTR. All the experiments were performed in triplicate. Quantitative data of the reporter gene assay are presented as mean ± SEM. Student's *t*-test was used to determine significant differences between two groups.

### Relative quantitative RT-PCR

RNA was isolated from HEK293T cells 24 hours post-transfection using TRIzol reagent according to the manufacturer's instructions (Life Technologies Inc., Ambion). Reverse transcription was performed using SSIII (Life Technologies Inc., Invitrogen, Burlington, ON, Canada). Relative quantitative PCR was performed by co-amplifying Firefly and Renilla luciferases encoded in the same pmirGLO vector using the following primers: fLuc: forward 5′ CAA GAC TAT AAG ATT CAA TCT GCC CTG CTG 3′ and reverse 5′ GAT GTT GGG GTG TTG CAG CAG GAT 3′; rLuc: forward 5′ GAG CAA CGC AAA CGC ATG ATC ACT G 3′ and reverse 5′ TTC AGC AGC TCG AAC CAA GCG GT 3′.

PCR products were run on 1% agarose gel and the intensity of bands was quantified by densitometry. Normalization of Firefly luciferase densitometry was performed against Renilla luciferase densitometry. Data were also normalized to the impact of each miRNA on the luciferase mRNA without *NEFL* mRNA 3′UTR and to the effect of miR-let-7a (miRNA control) on the luciferase mRNA to obtain the specific effect on the *NEFL* mRNA 3′UTR. All experiments were performed in triplicate. Student's *t*-test was used to determine significant differences between the two groups.

Semi-quantitative RT-PCR was also performed to observe the effect of each miRNA on endogenous *NEFL* mRNA in HEK293 T cells using the method described above. The following primers were used to amplify endogenous *NEFL* and GAPDH. GAPDH Forward: AATCTAGAGGGAGCCAAAAGGGTC, GAPDH Reverse: AGAATTCTCACGCCACAGTTTCC, *NEFL* Forward: CAAGACCCTGGAAATCGAAG, *NEFL* Reverse: TCTTGGACATGGCTGGTGTA.

### miRNA Inhibition Assay

Custom designed anti-mir-miRNA for mir-b2403, mir-b1336 and scramble control were purchased from Ambion (Life Technologies Inc., Applied Biosystems). HEK293 T cells were transfected with 3.47 fmol of pmirGLO-NEFL 3′UTR and pre-miRNAs using Lipofectamine 2000 reagent (Life Technologies Inc., Invitrogen, Burlington, ON, Canada) as described above. Cells were transfected with 100 nM anti-mir-miRNA inhibitors 24 hours post transfection. Twenty-four hours after anti-mir-miRNA transfection (total 48 hours after pre-mir-miRNAs and pmirGLO-*NEFL* 3′ UTR transfection), plates were read using luminometer or relative quantitative PCR performed as described above.

## Results

### Small RNA library from spinal cord

We previously used a commercially available miRNA array in order to identified a small number of known miRNAs that were differentially expressed between sALS and neuropathologically normal spinal cord tissue [40]. Recognizing that such commercial arrays are limited to known miRNAs, we have now generated a library of small RNAs from human spinal cord and analyzed the sequencing data from novel short RNA species that may be miRNAs. After sequencing, 80 putative novel miRNAs were detected. When analyzed for cross-species conservation, most were found to be either conserved only in primates or not conserved at all (Table S1). The library data is available for download at our website http://www.robarts.ca/strong/microRNA_Database/index.html.

### Putative novel miRNAs have MREs within the NEFL mRNA 3′UTR

To determine which of these putative novel miRNAs have recognition elements within the *NEFL* mRNA 3′UTR, we performed MRE prediction using the criterion of the seed sequence as outlined in the methods. *NEFL* mRNA was used as the target as our interest lies in understanding the mechanisms related to the selective suppression of NEFL mRNA in the ALS spinal cord motor neurons.

**Figure 1:**
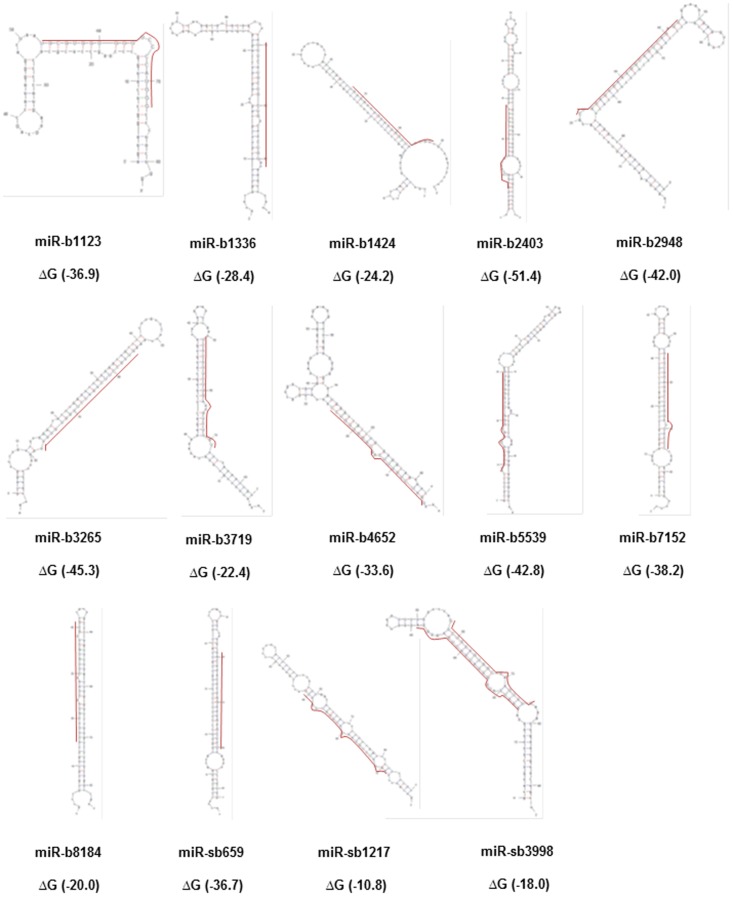
Predicted miRNA folding for putative novel miRNAs as determined using Mfold. Mature and/or star miRNA sequences are shown in capital letters and a line next to it for each hairpin precursor, when appropriate, according to expression. For pre-miR-sb659 and pre-miR-sb1217, only star sequences are highlighted, while for pre-miR-sb3998 both strands are in capital letters. ΔG (kcal/mol) for each hairpin structure is given in parentheses.

Of the 80 putative novel miRNAs detected in the spinal cord libraries, 24 had predicted MREs within the *NEFL* mRNA 3′UTR. We then used two online tools (see methods) to confirm miRNA structures. Using the Mfold, pre-miRNA folding structures were predicted and folding free energies (ΔGs) calculated. Several of the 24 predicted miRNAs did not pass the confirmation analysis using Computational Identification of microRNA (CID, Center for Computational Biology and Bioinformatics) [33] and mireval (http://tagc.univ-mrs.fr/mireval) [34] or passed but no satisfactory PCR primers could be custom designed from Ambion (Life Technologies Inc.) to allow additional study into expression and stability. Of the original 24, 15 putative miRNA that met these criteria and showed low folding free energies were included for further study (Table 2; Figure 1).

**Table 2:** Putative novel miRNAs that have predicted response elements [57] within the *NEFL* mRNA 3′UTR.

miRNA ID	Seed type^a^	Number of nucleotides in each detected MRE
miR-b1123	2	7
miR-b1336	1	7
miR-b1424	2	7
miR-b2403	1,3	7/7
miR-b2948	3	7
miR-b3265	3	8
miR-b3719	1	7
miRb4652	3	8
miR-b5539	2	7
miR-b7152	2	7
miR-b8184	1,2	8/7
miR-sb659*	1,2	8/7
miR-sb1217*	2	7
miR-sb3998	1,2	8/7/7/7^b^
miR-sb3998*	1,2	8/7

a. Beginning at position +1, +2 or +3 relative to the 5′-end of the miRNA

b. miR-sb3998 has 4 potential MREs (separated by slashes)

**Figure 2:**
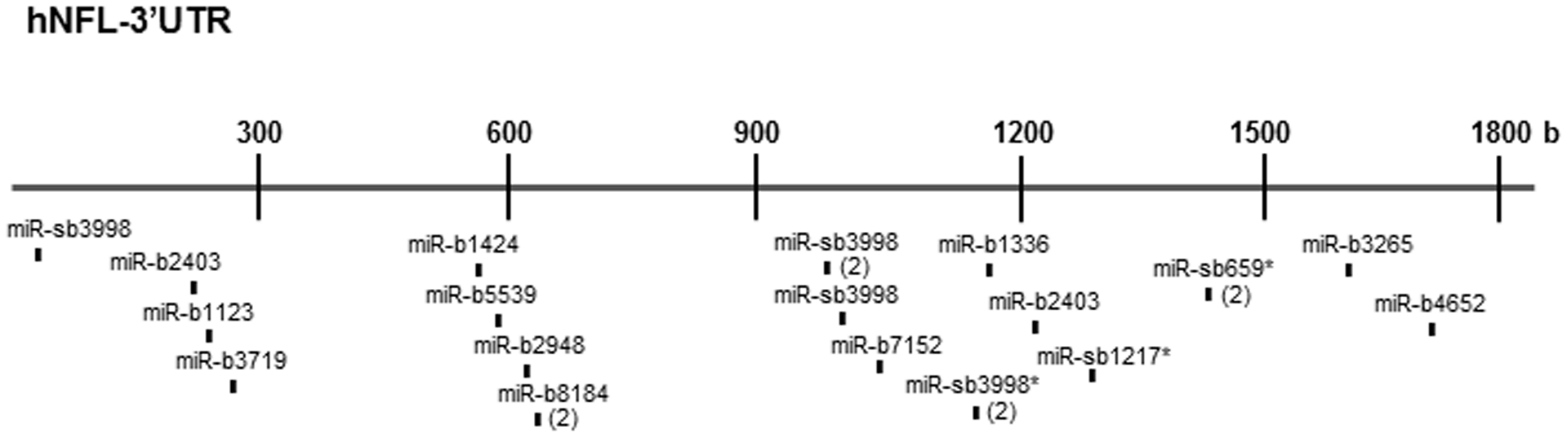
MiRNA response elements for the putative novel miRNAs in the *NEFL* mRNA 3′UTR. Note the presence of recognition sites for the many putative novel miRNAs throughout the 3′ UTR. In addition, some miRNAs have more than one MRE in different positions within the *NEFL* mRNA 3′UTR (e.g. miR-b2403) and others have more than one MRE in overlapped positions within the *NEFL* mRNA 3′UTR (e.g. miR-b8184; number of MREs in parentheses).

Most of these 15 novel miRNAs have a seed sequence beginning at position +1 or +2 relative to the 5′-end of the miRNA and one MRE of 7 nucleotides. However, some miRNAs had more than one MRE situated in different positions within the *NEFL* mRNA 3′UTR (e.g. miR-b2403), while others had more than one MRE in overlapped positions within the *NEFL* mRNA 3′UTR (e.g. miR-b8184; Figure 2).

These results indicate that there are novel miRNA expressed in the spinal cord that are predicted to target *NEFL* mRNA 3′UTR and that satisfy the criterion for being classified structurally as a miRNA.

### Differentially expressed miRNAs in sALS spinal cord

We then studied the expression levels of the novel miRNAs that fulfilled the above criteria with MREs in the *NEFL* mRNA 3′UTR in 5 sALS and 5 control spinal cord tissues using real-time PCR (table S2). Of the 15 novel miRNAs, 10 were differentially expressed. Compared to control tissues, miR-b1336, miR-b2403, miR-b4652 and miR-sb659* were down-regulated and miR-b1123, miR-b2948, miR-b3265, miR-b5539, miR-sb1217* and miR-sb3998 were up-regulated in spinal cord. The greatest down-regulation (26 fold decrease) was detected for miR-b1336 and miR-b4652 while the greatest up-regulation (18 fold increase) was observed for miR-sb1217* (Table 3).

**Table 3:** Putative novel miRNAs differentially expressed in sALS and control spinal cords.

miRNA ID	Log_10_RQ^a^	p Value
miR-b1123	1.556586	<0.001
miR-b1336	−2.6512	<0.001
miR-b2403	−0.99138	<0.01
miR-b2948	1.261971	<0.001
miR-b3265	0.1433	<0.001
miR-b4652	−2.67447	<0.1
miR-b5539	1.326557	<0.01
miR-sb659*	−0.72503	<0.001
miR-sb1217*	1.883788	<0.001
miR-sb3998	0.647787	<0.001

^a^ Negative values denote down-regulation, while positive values denote up-regulation of expression.

These results demonstrate that a group of novel miRNAs that have MREs within *NEFL* mRNA 3′UTR have differential expression in spinal cord in sALS compared to controls.

### Differentially expressed miRNAs influence endogenous NEFL expression in HEK293 T cells

**Figure 3:**
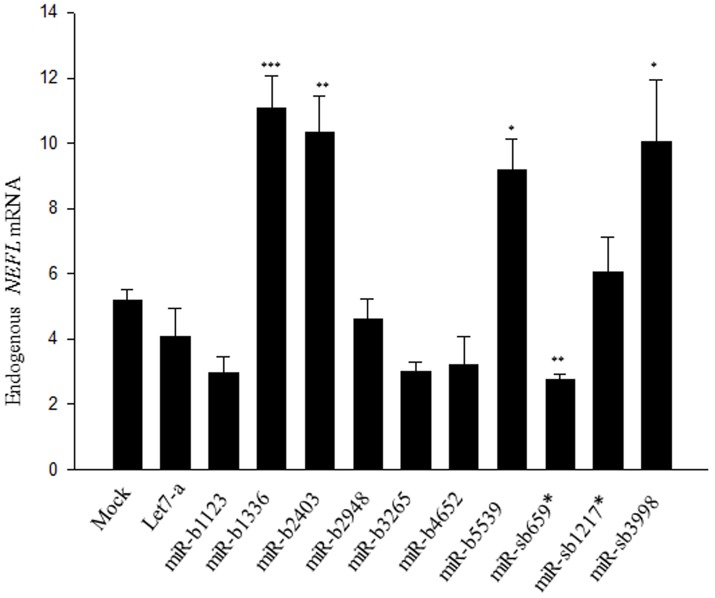
Effect of differentially expressed novel miRNAs on endogenous *NEFL* steady state mRNA levels in HEK293 T cells. Relative to mock (control), there was no significant change in the *NEFL* steady state mRNA levels for Let-7a (negative control), mir-b2948 or mir-sb1217*. Increased steady state levels were observed for mir-b1136, b2403, b5539 and sb3998. Reduced steady state levels were observed for mir-b1123, b3265, b4652 and sb659*. Results are shown as mean ± SEM (t-test: ***  = p<0.001; ** = p<0.005; * = p<0.05).

To study the effect of these dysregulated, differentially expressed novel miRNAs on endogenous *NEFL,* we transfected HEK293 T cells with pre-miRNAs and performed semi-quantitative RT-PCR for *NEFL* mRNA expression. Upregulation of *NEFL* mRNA was observed for mir-b1336, mir-b2403, mir-b5539 and mir-sb3998. *NEFL* mRNA was reduced when treated with mir-b1123, mir-b4652, mir-b3265 or mir-b659*. No significant change in endogenous *NEFL* mRNA expression was observed for mir-b1217*, mir-b2948 or control Let-7a (Figure 3). These results demonstrate that these miRNAs have capability to influence the expression of endogenous *NEFL* mRNA.

### Differentially expressed novel miRNAs regulate a reporter linked to the NEFL mRNA 3′UTR

To study whether novel miRNAs with altered expression in sALS can exert an effect through the predicted MREs found in the 3′UTR of *NEFL* mRNA, we used luciferase reporter assays and relative quantitative RT-PCR [40].

**Figure 4:**
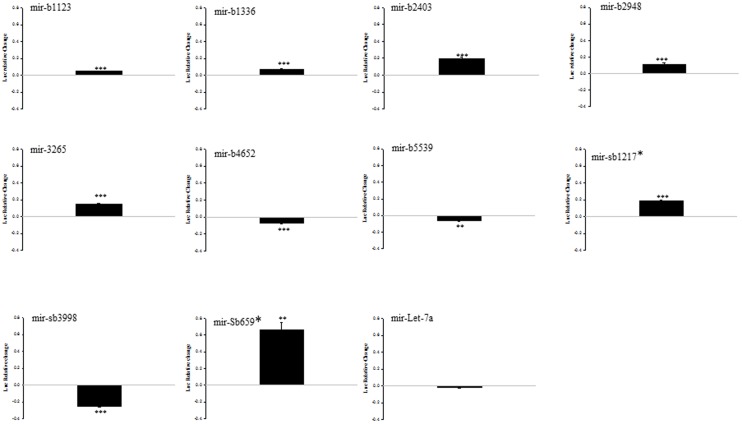
A group of novel miRNAs dysregulated in sALS regulates the activity of a reporter linked to *NEFL* 3′UTR. Luciferase reporter gene assay was performed by transfecting HEK293T cells with pre-miRNAs and a reporter construct expressing Firefly luciferase coupled to *NEFL* 3′UTR. Data are expressed as relative change compared to empty vector and show positive values as up-regulation and negative values as down-regulation. Experiments were performed in triplicate. Results are shown as mean ± SEM (*t*-test: p<0.001 = ***; p<0.005 = **).

In the reporter assay, we were interested in 1) novel miRNAs down-regulated in sALS that induced up-regulation of the Firefly luciferase activity and 2) novel miRNAs up-regulated in sALS that induced down-regulation of the Firefly luciferase activity. Both these conditions could result in the selective suppression of *NEFL* mRNA previously observed in sALS spinal motor neurons. Three miRNAs fulfilled the first criteria: miR-b1336, miR-b2403 and miR-sb659* induced a significant up-regulation of the Firefly luciferase activity (Figure 4) while the expression of each of these 3 miRNAs was down-regulated in sALS spinal cord (Table 3). Conversely, the expression levels of both miR-sb3998 and miR-b5539, each of which induced a significant down-regulation of the Firefly activity (Figure 4), was up-regulated in sALS (Table 3), thus fulfilling the second criteria.

**Figure 5:**
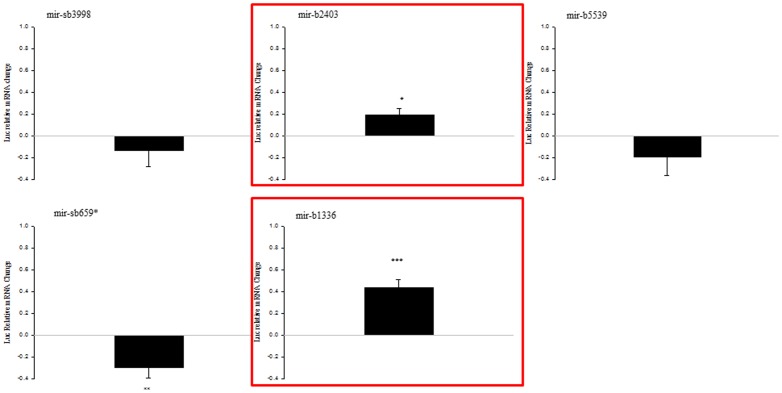
RT-PCR analysis of candidate miRNAs to study the effect on Firefly luciferase mRNA. MiRNA that showed opposite effects in the luciferase assay versus the RT-PCR data presented in Figure 4 and Table3. Two highlighted novel miRNAs that are down-regulated in sALS (mir-b1336 and mir-b2403; red boxes) regulate the expression of *NEFL* mRNA 3′UTR in a manner consistent with that predicted to lead to a decrease in *NEFL* mRNA. Relative quantitative RT-PCR was performed by transfecting HEK293T cells with pre-miRNAs and a reporter construct expressing Firefly luciferase coupled to the *NEFL* 3′UTR. Data are expressed as relative change compared to let-7a as control and show positive values as up-regulation and negative values as down-regulation. Experiments were performed in triplicate. Results are shown as mean ± SEM (t-test: p<0.001 = ***; p<0.005 = **; p<0.01 = *). No significant change was observed for mir-sb3998 and mir-b5539.

We then performed relative quantitative RT-PCR of the 5 miRNAs that showed opposite results in the reporter gene assay and real-time PCR to determine the effect of the miRNA on the expression of the Firefly luciferase mRNA and thus elucidate the action on stability via the 3′UTR. Only miR-b1336 and miR-b2403 increased the levels of Firefly luciferase mRNA bound to *NEFL* 3′UTR (Figure 5). These results are consistent with the up-regulation of the luciferase signal by miR-b1336 and miR-b2403. One would then predict that the down-regulation of the expression of these miRNA in sALS spinal cord then would lead to a decrease in the expression of the *NEFL* mRNA via lack of interaction with the 3′UTR.

The remaining novel miRNAs did not show a correlation between the results in the reporter gene assay, the relative quantitative RT-PCR, and the expression observed in sALS that could explain the suppression of *NEFL* mRNA observed in sALS spinal cord tissues (Figures 3 and 4). Given this, from amongst the original 24 miRNAs relevant to *NEFL* mRNA processing, only two (miR-b1336 and miR-b2403) could be considered as valid candidates for inducing the down-regulation of *NEFL* mRNA in sALS spinal cord using the stringent criteria described above.

### MiR-b1336 and miR-b2403 specifically regulate NEFL expression

**Figure 6:**
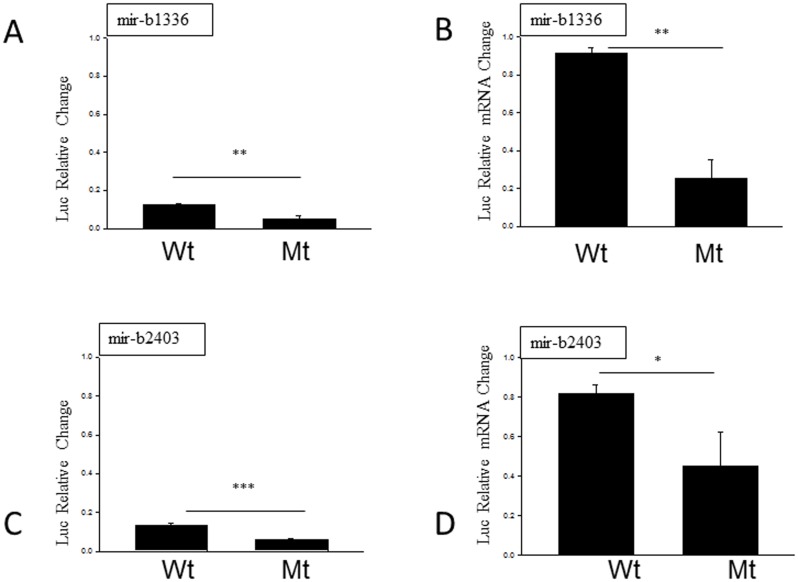
Mutations of the seed sequence of novel miRNAs (shown at Mt) compared to intact seed sequences (Wt) confirmed that both mir-b1336 and mir-b2403 interact directly with their respective seed sequences. A and C show the results of the luciferase reporter assay while B and D show the results from the RT-PCR analysis from mir-b1336 and mir-b2403, respectively. Data are expressed in terms of relative change and show up-regulation as positive values and down-regulation as negative values. Experiments were performed in triplicate. Results are shown as mean ± SEM (t-test: ***  = p<0.001; ** = p<0.005; * = p<0.05).

To confirm the specificity of miR-b1336 and miR-b2403, we used mutants of the seed sequences to confirm the direct effect of these two miRNAs on the regulation of the *NEFL* mRNA 3′ UTR. The luciferase reporter assays showed a significant decrease in activity and relative quantitative RT-PCR showed a significant decrease in luciferase mRNA expression when cells were transfected with mutants of miR-b1336 or miR-b2403 as opposed to the wild type in which the seed sequence was intact (Figure 6).

**Figure 7:**
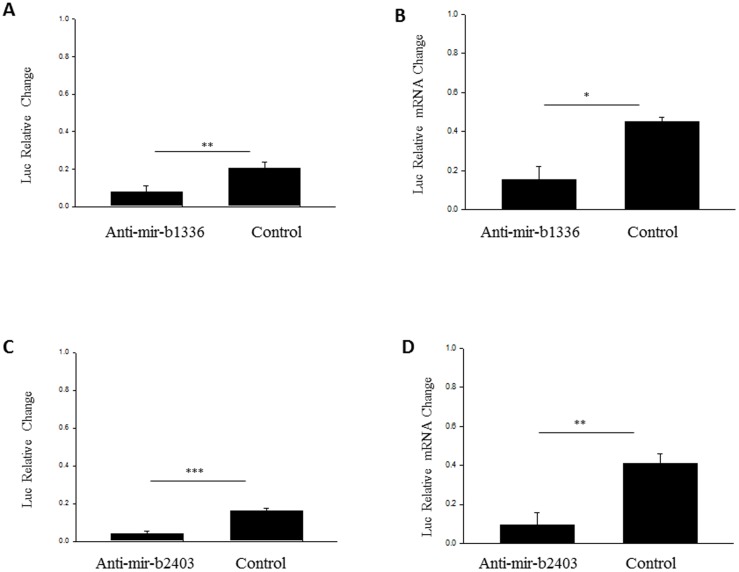
The inhibition of miR-b1336 and miR-b2403 using anti-miR-b136 and anti-miR-2403, respectively, resulted in the loss of the stabilizing effect. A and B represent the luciferase reporter assay and the RT-PCR analysis respectively from miR-b1336. Similarly, C and D represent luciferase reporter assay and the RT-PCR analysis for miR-b2403. Experiments were performed in triplicate and data are expressed as relative change. Results are shown as mean ± SEM (t-test: ***  = p<0.001; ** = p<0.005; * = p<0.05).

To confirm these findings, we next performed the reporter assay using anti-mir-miRNA inhibitors (Figure 7). Each significantly reduced the luciferase activity compared to control scramble. Using relative quantitative RT-PCR, we also observed a significant reduction in luciferase mRNA expression upon anti miR-b1336 and anti miR-b2403 treatment. *NEFL* mRNA expression was unaffected upon treatment with scramble control. Our results demonstrate that mutation of the seed sequence of the two novel miRNAs, miR-b1336 and miR-b2403, or their inhibition using anti-mir-miRNA inhibitors, prevents activity of these miRNA on the *NEFL* 3′UTR. This confirms that these two miRNA can specifically regulate the *NEFL* mRNA 3′UTR.

## Discussion

We have identified a group of novel miRNAs in human spinal cord tissue and from amongst these identified a panel of miRNAs with MREs in the human *NEFL* mRNA 3′UTR. From this group, the expression levels of 2, miR-b1336 and miR-b2403, were significantly down-regulated in sALS. Over expression of both of these novel miRNAs induced the up-regulation of the expression of a luciferase reporter bound to the *NEFL* mRNA 3′UTR. This effect could be fully inhibited using either mutations in the seed sequence (thus inhibiting direct interaction) or by the presence of anti-miR. We additionally proved that the effect of each could be observed on endogenous *NEFL* mRNA, thus providing evidence of an *in vivo* effect. It is thus reasonable to conclude that an increased activity or expression of either miR-b1336 or miR-b2403 would thus lead to increased *NEFL* mRNA stability. The corollary therefore is that the loss of either, as observed in ALS spinal cord, would be expected to contribute directly to the reduction in NEFL steady state mRNA level that is pathognomic of ALS spinal motor neurons.

While the conventional view is that miRNA mediate mRNA decay or translation repression [41], recent evidence suggests that miRNA may also stabilize mRNA [27] and enhance translation [28]. There are in fact a number of miRNA in which the association with either the 3′ or 5′ UTR leads to target mRNA translational activation rather than repression, a topic that has been recently reviewed in great detail [42], [43]. This includes stabilization of Hepatitis C virus RNA by an Ago2-miR-122 complex [27], reversal of translational repression by the association of miRNA-10a binding to the 5′UTR of ribosomal protein mRNAs and enhancing translation [28], and a significant translational upregulation of *Myocad* as assayed in a luciferase assay identical to that performed in our studies when co-transfected with miR-145 [44]. The ability of a single miRNA to act in both a repressive and activating manner dependant on the extent of base pairing or the cell cycle stage has been well described [45], [46], [47], [48]. Moreover, this can be further modulated by the presence of alternative polyadenylated variants as described for β-actin [26]. A key role for the length of the poly(A) tail interacting with the Ago proteins in determining whether a mRNA undergoes translational activation or repression, governed by the miRNA, has also been described [49]. The mechanisms that underlie the post-transcriptional stimulation of gene expression by miRNAs is particularly well laid out by Lee and Vasuden, including the direct effect upon the 3′UTR as described in our paper [50]. Our previous work also showed that miR-524-5p and miR-582-3p induce an up-regulation of reporter bound to *NEFL* mRNA 3′UTR [40]. Hence, while the concept of miRNA-mediated translational up-regulation is recent, there is significant support for our findings.

The postulate that miRNAs participate in the neurodegenerative process of ALS has been supported by a limited number of studies, including inferences from murine models of motor neuron degeneration. This includes the observation that the expression of the skeletal muscle specific miRNA miR-206 is up-regulated early in the course of the disease process in the G93A mutant superoxide dismutase (SOD1) model of familial ALS, consistent with a role in response to injury of the neuromuscular junction [51]. Haramati and colleagues [52] have demonstrated in a murine model of progressive motor neuron disease that neuron-specific miR-9, the most abundant miRNA in murine motor neurons, is selectively down-regulated thus allowing for a selective increase in the high molecular weight NF (*NEFH*) mRNA relative to either *NEFL* or *NEFM*. Using a commercially available array to examine miRNA expression in the frontal cortex of ALS patients, Shioya and colleagues [53] observed an up-regulation of miR-29a, miR-29b and miR-338-39. The latter authors elected to further study only miR-29a and miR-338-3p. While neither was expressed at significantly different levels in ALS tissues when contrasted to a control, it is of note that when examined in Alzheimer's frontal cortex, miR-338-3p expression was significantly up-regulated. The same miRNA (miR-338-3p) was subsequently observed to be elevated in blood (amongst a panel of 8 in total which were elevated) from ALS patients compared to healthy control [54]. More recently, using a commercially available array to examine miRNA expression in ventral spinal cord from ALS patients, we observed that the expression of the majority of miRNAs was reduced in ALS, although several were differentially expressed between ALS and controls [40]. Three (miR-146a*, miR-524-5p and miR-582-3p) were capable of interacting with *NEFL* mRNA in a manner consistent with the selective reduction of *NEFL* mRNA.

All these studies have shown that the expression of a variety of miRNAs are altered in ALS and suggests a potential role in its pathogenicity. Our previous work has demonstrated a role for RNA species in modulating the stability of the *NEFL* transcript in spinal cord in ALS [19] and we have observed that *NEFL* mRNA differentially partitions to processing bodies in ALS spinal motor neurons [19], again supporting the notion of miRNA being involved in *NEFL* mRNA processing. These results lead to our current hypothesis that the differential expression of miRNAs in ALS is a key determinant of the stability of *NEFL* mRNA in ALS and that this contributes directly to the altered stoichiometry of NF mRNA expression observed in ALS spinal motor neurons.

This current study is complementary to our earlier work [33] in that we have now generated a small RNA library for sALS and control spinal cord and from this characterized previously unrecognized miRNAs. We found 80 putative novel miRNAs of which 15 were confirmed structurally to match the criteria to be miRNAs involved in the regulation of *NEFL* mRNA. Most of these latter novel miRNAs showed low folding free energies. It has been reported that in contrast with transfer RNAs and ribosomal RNAs, miRNAs exhibit considerably lower ΔGs than shuffled sequences. This indicates a high tendency in the sequence to form a stable secondary structure [55]. In fact, one of the two novel miRNAs candidates we obtained for the regulation of *NEFL* mRNA (miR-b2403) showed the lowest ΔG in the entire group.

Our expression studies by real-time PCR showed that some novel miRNAs which have MREs in the *NEFL* mRNA 3′UTR are down-regulated while others are up-regulated in sALS spinal cord tissue. The expression levels of our final candidates, miR-b1336 and miR-b2403, are both down-regulated in sALS. MiR-b1336 has one MRE in the *NEFL* mRNA 3′UTR while miR-b2403 has two MREs in distant positions within the 3′UTR. In unaffected spinal cord it is possible that these two miRNA may function either independently or in an additive manner to regulate stability of the *NEFL* transcript. It would follow then that in sALS, the decrease in their expression may cause the loss of the transcript through failure of stabilizing or increasing transcription of *NEFL* mRNA. Additionally, functional analysis by reporter gene assay and relative quantitative RT-PCR showed that both miR-b1336 and miR-b2403 up-regulate the expression of the luciferase bound to *NEFL* mRNA 3′UTR. Predominantly, miRNAs act as post-transcriptional gene regulators that result in mRNA decay or translational silencing, though miRNAs can also have an up-regulatory effect on mRNA expression. For example, one miRNA could mask a critical interaction region of a destabilizing protein changing the net effect on the mRNA's stability and resulting in up-regulation of expression.

Together with our previous observations, we have now identified a pool of miRNAs which are differentially expressed in sALS spinal cord tissue and which collectively lead to the loss of *NEFL* mRNA stability. Experiments to understand whether this panel of miRNAs acts co-operatively or not are ongoing. However, the mechanism of action of miRNAs, acting individually or cooperatively, and using one or more MREs in the target, is becoming more complex with the emergence of competing endogenous RNAs (termed “miRNA sponges”) [56]. Further defining the impact of interaction of miRNA (or lack of a normal interaction) on the regulation of their targets, the mechanism by which they act (possibly through allowing or disallowing other RNA stability factor interaction), and the net effect of all the stability factors that are interacting with a transcript at any given point will be necessary to understanding the role of RNA metabolism and how this is altered in the ALS disease state.

## Supporting Information

Figure S1 **Composition of the libraries.**
(TIFF)

Table S1 **Putative novel miRNAs expressed in the human spinal cord.**
(DOCX)

Table S2 **Patient demographics of samples used in real-time PCR analyses.**
(DOCX)
