# Supplementary figures and images for: Analysis of Novel NEFL mRNA Targeting microRNAs in Amyotrophic Lateral Sclerosis
Source: PLoS One. 2014 Jan 15;9(1):e85653. doi: 10.1371/journal.pone.0085653 (PMC3893244; doi:10.1371/journal.pone.0085653)

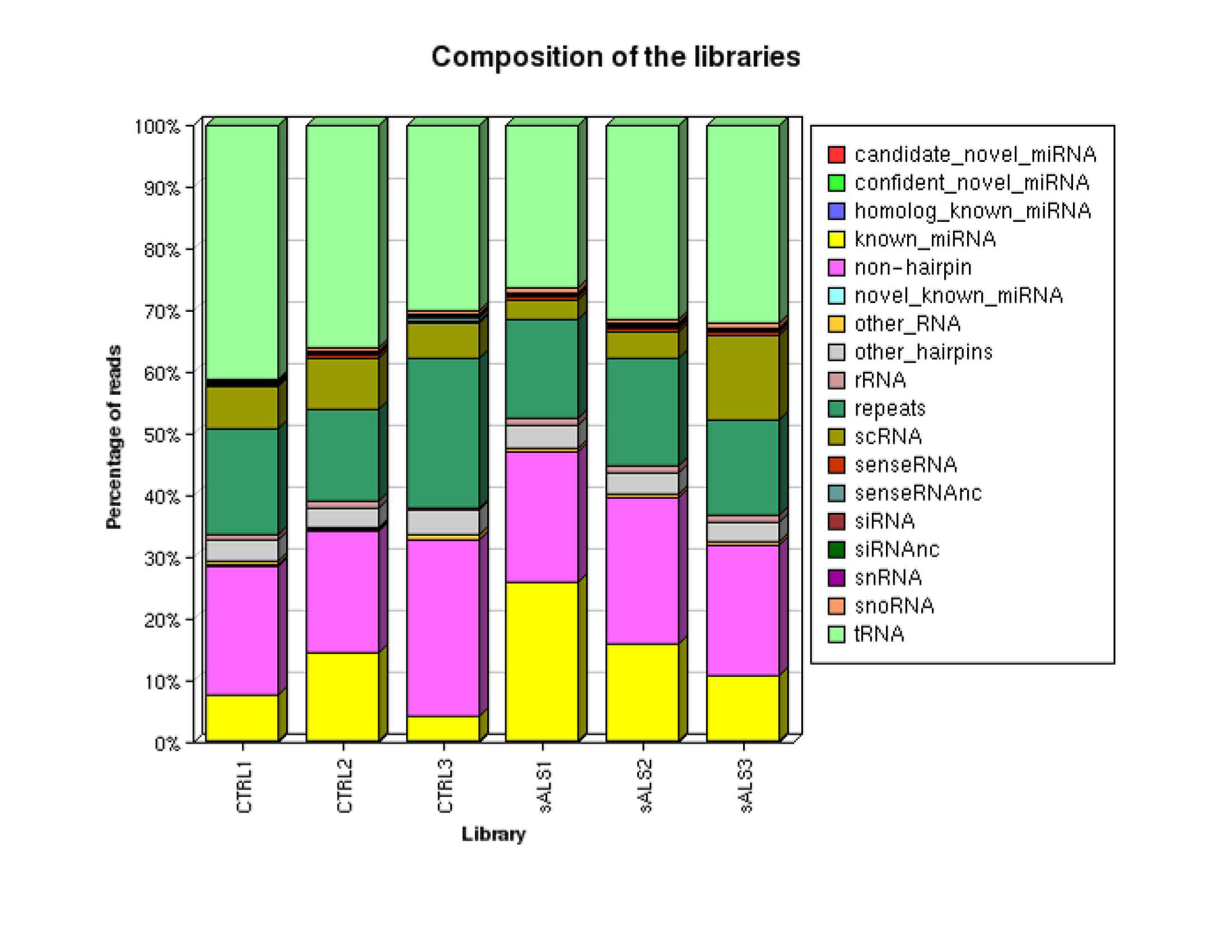

Supplement: Figure S1 — Composition of the libraries. (TIFF) [file pone.0085653.s001.tif]
