# Supplementary material for: Analysis of Novel NEFL mRNA Targeting microRNAs in Amyotrophic Lateral Sclerosis
Source: PLoS One. 2014 Jan 15;9(1):e85653. doi: 10.1371/journal.pone.0085653 (PMC3893244; doi:10.1371/journal.pone.0085653)
Supplement: Table S1 — Putative novel miRNAs expressed in the human spinal cord. (DOCX) [file pone.0085653.s002.docx]

**Table S1.** Putative novel miRNAs expressed in human spinal cord

| **miR ID** | **Mature sequence 5’-3’** | **Mature star sequence 5’-3’** | **Chr** | **Conserved species** |
| --- | --- | --- | --- | --- |
| miR-[b107](file:///C:\Users\Dani\Desktop\DANAE%2029-01-13\Strong%20Lab\Strong%20Lab%20papers\miRNAs%20nuevos\expression\miR\block10798878_novel.html)9 | GGUCCCAGGCUAGGUAGACAUGG | - | 8 | - |
| miR-[b108](file:///C:\Users\Dani\Desktop\DANAE%2029-01-13\Strong%20Lab\Strong%20Lab%20papers\miRNAs%20nuevos\expression\miR\block10855294_novel.html)5 | AAGGCAAUAUUGUGGCAUUAUU | - | 8 | chimp |
| [miR-b110](file:///C:\Users\Dani\Desktop\DANAE%2029-01-13\Strong%20Lab\Strong%20Lab%20papers\miRNAs%20nuevos\expression\miR\block11023520_novel.html)2 | AAUGCAUGUCCCAGUCUUCUCU | - | 9 | chimp |
| [miR-b111](file:///C:\Users\Dani\Desktop\DANAE%2029-01-13\Strong%20Lab\Strong%20Lab%20papers\miRNAs%20nuevos\expression\miR\block11168710_novel.html)6 | AGGAAUAGUCUUCAGCACUUU | - | 9 | chimp, macaque |
| [miR-b112](file:///C:\Users\Dani\Desktop\DANAE%2029-01-13\Strong%20Lab\Strong%20Lab%20papers\miRNAs%20nuevos\expression\miR\block11232983_novel.html)3 | UGCGUGGUGGGUCCCCAUGGG | - | 9 | - |
| [miR-b118](file:///C:\Users\Dani\Desktop\DANAE%2029-01-13\Strong%20Lab\Strong%20Lab%20papers\miRNAs%20nuevos\expression\miR\block11865845_novel.html)6 | CCCAUCUCCACCUGGACCCAG | - | X | - |
| [miR-b132](file:///C:\Users\Dani\Desktop\DANAE%2029-01-13\Strong%20Lab\Strong%20Lab%20papers\miRNAs%20nuevos\expression\miR\block1326139_novel.html)6 | GCCACGGGGGGGCCUGGCCACG | - | 12 | - |
| [miR-b133](file:///C:\Users\Dani\Desktop\DANAE%2029-01-13\Strong%20Lab\Strong%20Lab%20papers\miRNAs%20nuevos\expression\miR\block1336549_novel.html)6 | AUUUCAUGCUGCUGACAACUGUU | - | 12 | chimp |
| [miR-b142](file:///C:\Users\Dani\Desktop\DANAE%2029-01-13\Strong%20Lab\Strong%20Lab%20papers\miRNAs%20nuevos\expression\miR\block1424085_novel.html)4 | UGAAGAAAGAAAAGGUUAAUGC | - | 12 | macaque |
| [miR-b144](file:///C:\Users\Dani\Desktop\DANAE%2029-01-13\Strong%20Lab\Strong%20Lab%20papers\miRNAs%20nuevos\expression\miR\block1444725_novel.html)4 | AACACUGGCCUUGCUAUCCCCA | - | 12 | mouse |
| [miR-b144](file:///C:\Users\Dani\Desktop\DANAE%2029-01-13\Strong%20Lab\Strong%20Lab%20papers\miRNAs%20nuevos\expression\miR\block1447442_novel.html)7 | CCUCUGGGCCUCCUUUUUCCAGG | - | 12 | chimp, macaque |
| [miR-b157](file:///C:\Users\Dani\Desktop\DANAE%2029-01-13\Strong%20Lab\Strong%20Lab%20papers\miRNAs%20nuevos\expression\miR\block1574195_novel.html)4 | ACAUUUGUAGGGGCUGGAGGGU | - | 12 | chimp, macaque |
| [miR-b160](file:///C:\Users\Dani\Desktop\DANAE%2029-01-13\Strong%20Lab\Strong%20Lab%20papers\miRNAs%20nuevos\expression\miR\block1602161_novel.html)2 | GUGCCCACUCCCCGACACCUCG | - | 12 | chimp, macaque |
| [miR-b170](file:///C:\Users\Dani\Desktop\DANAE%2029-01-13\Strong%20Lab\Strong%20Lab%20papers\miRNAs%20nuevos\expression\miR\block1701693_novel.html)1 | GCUGUGGUAAACUUUACGAUUC | - | 13 | chimp, macaque |
| [miR-b200](file:///C:\Users\Dani\Desktop\DANAE%2029-01-13\Strong%20Lab\Strong%20Lab%20papers\miRNAs%20nuevos\expression\miR\block2006624_novel.html)6 | AACAAAGUUGCGACAUAUGACAG | - | 13 |  |
| [miR-b212](file:///C:\Users\Dani\Desktop\DANAE%2029-01-13\Strong%20Lab\Strong%20Lab%20papers\miRNAs%20nuevos\expression\miR\block2122743_novel.html)2 | AGACACAAGUUGGCCACUCUG | - | 14 | chimp |
| [miR-b218](file:///C:\Users\Dani\Desktop\DANAE%2029-01-13\Strong%20Lab\Strong%20Lab%20papers\miRNAs%20nuevos\expression\miR\block2188362_novel.html)8 | GCCCAGCACUGAGUCAUUGGCU | - | 14 | chimp, mouse |
| [miR-b224](file:///C:\Users\Dani\Desktop\DANAE%2029-01-13\Strong%20Lab\Strong%20Lab%20papers\miRNAs%20nuevos\expression\miR\block2249916_novel.html)9 | UCCCUAGUGGUCUACACCAUCUUU | - | 14 | - |
| [miR-b240](file:///C:\Users\Dani\Desktop\DANAE%2029-01-13\Strong%20Lab\Strong%20Lab%20papers\miRNAs%20nuevos\expression\miR\block2403141_novel.html)3 | GCAGCAAUGACUCAGUCGGGCU | - | 14 | chimp |
| [miR-b250](file:///C:\Users\Dani\Desktop\DANAE%2029-01-13\Strong%20Lab\Strong%20Lab%20papers\miRNAs%20nuevos\expression\miR\block250208_novel.html)2 | CCAAGGCGUGGCAAGUGGCCUG | - | 10 | chimp |
| [miR-b255](file:///C:\Users\Dani\Desktop\DANAE%2029-01-13\Strong%20Lab\Strong%20Lab%20papers\miRNAs%20nuevos\expression\miR\block2556562_novel.html)6 | CCAGGCAUCCGAAUCAUCCCA | - | 15 | chimp, macaque |
| [miR-b256](file:///C:\Users\Dani\Desktop\DANAE%2029-01-13\Strong%20Lab\Strong%20Lab%20papers\miRNAs%20nuevos\expression\miR\block2566962_novel.html)6 | GCCUACUGUCAGGUCAGCACG | - | 15 | chimp, macaque |
| [miR-b280](file:///C:\Users\Dani\Desktop\DANAE%2029-01-13\Strong%20Lab\Strong%20Lab%20papers\miRNAs%20nuevos\expression\miR\block2802526_novel.html)2 | UAUAACGUGGUUACUGUAGCUCA | - | 16 | - |
| [miR-b287](file:///C:\Users\Dani\Desktop\DANAE%2029-01-13\Strong%20Lab\Strong%20Lab%20papers\miRNAs%20nuevos\expression\miR\block2879067_novel.html)9 | GGAGGUUGGGUAGGACUUGGGAGA | - | 16 | chimp |
| [miR-b291](file:///C:\Users\Dani\Desktop\DANAE%2029-01-13\Strong%20Lab\Strong%20Lab%20papers\miRNAs%20nuevos\expression\miR\block2915298_novel.html)5 | UUCCCACUGUCCCUACCUCCCU | - | 16 | - |
| [miR-b294](file:///C:\Users\Dani\Desktop\DANAE%2029-01-13\Strong%20Lab\Strong%20Lab%20papers\miRNAs%20nuevos\expression\miR\block2945770_novel.html)5 | AGAGGACUCUGGGAUGGAGUGUCCG | - | 16 | - |
| [miR-b294](file:///C:\Users\Dani\Desktop\DANAE%2029-01-13\Strong%20Lab\Strong%20Lab%20papers\miRNAs%20nuevos\expression\miR\block2948894_novel.html)8 | CUGGAGAGAAAGGCAGUCAGAGGA | - | 16 | chimp |
| [miR-b326](file:///C:\Users\Dani\Desktop\DANAE%2029-01-13\Strong%20Lab\Strong%20Lab%20papers\miRNAs%20nuevos\expression\miR\block3265659_novel.html)5 | GUAGCCUUCAGAUCUUGGUGUUUU | - | 17 | chimp |
| [miR-b329](file:///C:\Users\Dani\Desktop\DANAE%2029-01-13\Strong%20Lab\Strong%20Lab%20papers\miRNAs%20nuevos\expression\miR\block3298966_novel.html)8 | UUAUGUGUGCAUGUGCUUGGG | - | 17 | - |
| [miR-b335](file:///C:\Users\Dani\Desktop\DANAE%2029-01-13\Strong%20Lab\Strong%20Lab%20papers\miRNAs%20nuevos\expression\miR\block3358190_novel.html)8 | CACUUAUAAAAAUUUCCAAUUA | - | 17 | - |
| [miR-b346](file:///C:\Users\Dani\Desktop\DANAE%2029-01-13\Strong%20Lab\Strong%20Lab%20papers\miRNAs%20nuevos\expression\miR\block3469157_novel.html)9 | GAAUGGCAGCAGGGGUUGGGGAA | - | 17 | chimp, macaque |
| [miR-b369](file:///C:\Users\Dani\Desktop\DANAE%2029-01-13\Strong%20Lab\Strong%20Lab%20papers\miRNAs%20nuevos\expression\miR\block3699096_novel.html)9 | UGGCUCACAAGCUGAGGGUGUG | - | 18 | chimp |
| [miR-b371](file:///C:\Users\Dani\Desktop\DANAE%2029-01-13\Strong%20Lab\Strong%20Lab%20papers\miRNAs%20nuevos\expression\miR\block3719239_novel.html)9 | UCACAUUCUUGAAAGUCUUGU | - | 18 |  |
| [miR-b382](file:///C:\Users\Dani\Desktop\DANAE%2029-01-13\Strong%20Lab\Strong%20Lab%20papers\miRNAs%20nuevos\expression\miR\block3828960_novel.html)8 | ACAGCCCGGAUCCCAGCCCACUUAC | - | 19 | chimp, mouse |
| [miR-b404](file:///C:\Users\Dani\Desktop\DANAE%2029-01-13\Strong%20Lab\Strong%20Lab%20papers\miRNAs%20nuevos\expression\miR\block404235_novel.html)2 | GGUUUGUAGGAGUCAGUUCCUG | - | 10 | chimp |
| [miR-b407](file:///C:\Users\Dani\Desktop\DANAE%2029-01-13\Strong%20Lab\Strong%20Lab%20papers\miRNAs%20nuevos\expression\miR\block4072884_novel.html)2 | CGGCGGCGAGGGAGGUGGGGGGG | - | 19 | chimp, macaque |
| [miR-b410](file:///C:\Users\Dani\Desktop\DANAE%2029-01-13\Strong%20Lab\Strong%20Lab%20papers\miRNAs%20nuevos\expression\miR\block4102951_novel.html)2 | CCCUGGUCCCUCCCAUCACCCAC | - | 1 | macaque |
| [miR-b414](file:///C:\Users\Dani\Desktop\DANAE%2029-01-13\Strong%20Lab\Strong%20Lab%20papers\miRNAs%20nuevos\expression\miR\block4147712_novel.html)7 | UUUCGAUGGUAGUAAAAUGGA | - | 1 |  |
| [miR-b433](file:///C:\Users\Dani\Desktop\DANAE%2029-01-13\Strong%20Lab\Strong%20Lab%20papers\miRNAs%20nuevos\expression\miR\block43383_novel.html)8 | AGGGACUCUGGAGCCAUGUAU | - | 10 | chimp, macaque |
| [miR-b436](file:///C:\Users\Dani\Desktop\DANAE%2029-01-13\Strong%20Lab\Strong%20Lab%20papers\miRNAs%20nuevos\expression\miR\block4365854_novel.html)5 | GCCUCAGGCUCGCCUCGCCCA | - | 1 | - |
| [miR-b443](file:///C:\Users\Dani\Desktop\DANAE%2029-01-13\Strong%20Lab\Strong%20Lab%20papers\miRNAs%20nuevos\expression\miR\block4435613_novel.html)5 | CUUCAGGAAUCCCAAAAGCAGCA | - | 1 | - |
| [miR-b465](file:///C:\Users\Dani\Desktop\DANAE%2029-01-13\Strong%20Lab\Strong%20Lab%20papers\miRNAs%20nuevos\expression\miR\block4652280_novel.html)2 | UGAUCUGUUUCAGCAGGUGGUCC | - | 1 | mouse |
| [miR-b465](file:///C:\Users\Dani\Desktop\DANAE%2029-01-13\Strong%20Lab\Strong%20Lab%20papers\miRNAs%20nuevos\expression\miR\block4656664_novel.html)6 | CAGUGCUUAUCAUCCAGUGGGA | - | 1 | - |
| [miR-b499](file:///C:\Users\Dani\Desktop\DANAE%2029-01-13\Strong%20Lab\Strong%20Lab%20papers\miRNAs%20nuevos\expression\miR\block4991050_novel.html)1 | GGCUGCAGGCUAGGCUGGCCCUUC | - | 1 | - |
| [miR-b532](file:///C:\Users\Dani\Desktop\DANAE%2029-01-13\Strong%20Lab\Strong%20Lab%20papers\miRNAs%20nuevos\expression\miR\block5323516_novel.html)3 | CUUUCAACUAACGUGGGAACUGA | - | 20 | - |
| [miR-b533](file:///C:\Users\Dani\Desktop\DANAE%2029-01-13\Strong%20Lab\Strong%20Lab%20papers\miRNAs%20nuevos\expression\miR\block5335194_novel.html)5 | UGUGCUGGCCUCAAUGGGGGUGA | - | 20 | - |
| [miR-b538](file:///C:\Users\Dani\Desktop\DANAE%2029-01-13\Strong%20Lab\Strong%20Lab%20papers\miRNAs%20nuevos\expression\miR\block5389445_novel.html)9 | UCCCUGAGAAGGAUUGGUCUUG | - | 21 | - |
| [miR-b553](file:///C:\Users\Dani\Desktop\DANAE%2029-01-13\Strong%20Lab\Strong%20Lab%20papers\miRNAs%20nuevos\expression\miR\block5539544_novel.html)9 | UCCUCAGAGUGGGCUGGGUGUG | - | 22 | - |
| [miR-b579](file:///C:\Users\Dani\Desktop\DANAE%2029-01-13\Strong%20Lab\Strong%20Lab%20papers\miRNAs%20nuevos\expression\miR\block579089_novel.html)0 | CAGCAGUUUUCCCAGGUUAUC | - | 11 | - |
| [miR-b588](file:///C:\Users\Dani\Desktop\DANAE%2029-01-13\Strong%20Lab\Strong%20Lab%20papers\miRNAs%20nuevos\expression\miR\block5889950_novel.html)9 | CCCCGGUGUUGGGGCGCGUCUG | - | 2 | chimp, macaque |
| [miR-b635](file:///C:\Users\Dani\Desktop\DANAE%2029-01-13\Strong%20Lab\Strong%20Lab%20papers\miRNAs%20nuevos\expression\miR\block6358787_novel.html)8 | AUGUACCUCAGGGGCUUGUGC | - | 2 | - |
| [miR-b668](file:///C:\Users\Dani\Desktop\DANAE%2029-01-13\Strong%20Lab\Strong%20Lab%20papers\miRNAs%20nuevos\expression\miR\block6683569_novel.html)3 | UGGGGUGUCCAGCUCACAGGC | - | 3 | - |
| [miR-b689](file:///C:\Users\Dani\Desktop\DANAE%2029-01-13\Strong%20Lab\Strong%20Lab%20papers\miRNAs%20nuevos\expression\miR\block6895817_novel.html)5 | CAGAGUGUGGCUUGGUGUGGGGCC | - | 3 | chimp, macaque |
| [miR-b704](file:///C:\Users\Dani\Desktop\DANAE%2029-01-13\Strong%20Lab\Strong%20Lab%20papers\miRNAs%20nuevos\expression\miR\block7043946_novel.html)3 | UAAAACAUGGAAGUCAUUGGC | - | 3 | - |
| [miR-b715](file:///C:\Users\Dani\Desktop\DANAE%2029-01-13\Strong%20Lab\Strong%20Lab%20papers\miRNAs%20nuevos\expression\miR\block7152898_novel.html)2 | GAGUGGAGGCUAGGAUCACCC | - | 3 | - |
| [miR-b720](file:///C:\Users\Dani\Desktop\DANAE%2029-01-13\Strong%20Lab\Strong%20Lab%20papers\miRNAs%20nuevos\expression\miR\block7202781_novel.html)2 | AGUCCCACAACACUUAGUGUA | - | 3 | - |
| [miR-b720](file:///C:\Users\Dani\Desktop\DANAE%2029-01-13\Strong%20Lab\Strong%20Lab%20papers\miRNAs%20nuevos\expression\miR\block7205552_novel.html)5 | UUCUUGCUGUGGUUGCAUGUCU | - | 3 | - |
| [miR-b725](file:///C:\Users\Dani\Desktop\DANAE%2029-01-13\Strong%20Lab\Strong%20Lab%20papers\miRNAs%20nuevos\expression\miR\block7252816_novel.html)2 | CUAGAAGAUCUGUGACUUCAG | - | 3 | chimp |
| [miR-b773](file:///C:\Users\Dani\Desktop\DANAE%2029-01-13\Strong%20Lab\Strong%20Lab%20papers\miRNAs%20nuevos\expression\miR\block7730007_novel.html)0 | UACCUGGUAACUCAACUUGUAG | - | 4 | chimp |
| [miR-b800](file:///C:\Users\Dani\Desktop\DANAE%2029-01-13\Strong%20Lab\Strong%20Lab%20papers\miRNAs%20nuevos\expression\miR\block8004544_novel.html)4 | GUGGGAACAAUUACUGUUGGUU | - | 4 | chicken, chimp, macaque, mouse |
| [miR-b818](file:///C:\Users\Dani\Desktop\DANAE%2029-01-13\Strong%20Lab\Strong%20Lab%20papers\miRNAs%20nuevos\expression\miR\block8184170_novel.html)4 | AUUUUUGUCACUAAAUUUGUGCUUUG | - | 4 | - |
| [miR-b820](file:///C:\Users\Dani\Desktop\DANAE%2029-01-13\Strong%20Lab\Strong%20Lab%20papers\miRNAs%20nuevos\expression\miR\block8207683_novel.html)7 | AACCUUAUUCUUAAUGCCUUG | - | 5 | - |
| [miR-b845](file:///C:\Users\Dani\Desktop\DANAE%2029-01-13\Strong%20Lab\Strong%20Lab%20papers\miRNAs%20nuevos\expression\miR\block8456091_novel.html)6 | GGUGCUGUCUGAGCUGACAUGUG | - | 5 | chimp, macaque |
| [miR-b864](file:///C:\Users\Dani\Desktop\DANAE%2029-01-13\Strong%20Lab\Strong%20Lab%20papers\miRNAs%20nuevos\expression\miR\block8649708_novel.html)9 | UCUUUGACCGCUGUCCUGUACUC | - | 5 | chimp |
| [miR-b873](file:///C:\Users\Dani\Desktop\DANAE%2029-01-13\Strong%20Lab\Strong%20Lab%20papers\miRNAs%20nuevos\expression\miR\block8730343_novel.html)0 | UUAUCUGACUCAGCAGGACAC | - | 5 | chimp |
| [miR-b880](file:///C:\Users\Dani\Desktop\DANAE%2029-01-13\Strong%20Lab\Strong%20Lab%20papers\miRNAs%20nuevos\expression\miR\block8805449_novel.html)5 | AGGGGACAUCUAGGCUGUCCAGA | - | 5 | chimp, macaque |
| [miR-b939](file:///C:\Users\Dani\Desktop\DANAE%2029-01-13\Strong%20Lab\Strong%20Lab%20papers\miRNAs%20nuevos\expression\miR\block9394826_novel.html)4 | UCCCUUGUCUCCUUUCCCUAGCUUG | - | 6 | - |
| [miR-b954](file:///C:\Users\Dani\Desktop\DANAE%2029-01-13\Strong%20Lab\Strong%20Lab%20papers\miRNAs%20nuevos\expression\miR\block9542163_novel.html)2 | GGAAUAGUAGUUAUACUCUGU | - | 6 | chicken |
| [miR-b968](file:///C:\Users\Dani\Desktop\DANAE%2029-01-13\Strong%20Lab\Strong%20Lab%20papers\miRNAs%20nuevos\expression\miR\block9686255_novel.html)6 | UGAGGGCUGCUGGGGGACGGUC | - | 7 | - |
| [miR-b987](file:///C:\Users\Dani\Desktop\DANAE%2029-01-13\Strong%20Lab\Strong%20Lab%20papers\miRNAs%20nuevos\expression\miR\block9875206_novel.html)5 | GUGCCAACAGCAUGCUGGGGGC | - | 7 | macaque, mouse |
| [miR-b989](file:///C:\Users\Dani\Desktop\DANAE%2029-01-13\Strong%20Lab\Strong%20Lab%20papers\miRNAs%20nuevos\expression\miR\block9895463_novel.html)5 | UCCCUUCUCACCUAACUCAUGCUC | - | 7 | chimp |
| [miR-b989](file:///C:\Users\Dani\Desktop\DANAE%2029-01-13\Strong%20Lab\Strong%20Lab%20papers\miRNAs%20nuevos\expression\miR\block9897876_novel.html)7 | GAAAGUUCUGGGAUUGACUCA | - | 7 | chimp |
| [miR-sb463](file:///C:\Users\Dani\Desktop\DANAE%2029-01-13\Strong%20Lab\Strong%20Lab%20papers\miRNAs%20nuevos\expression\miR\sblock463_novel.html) | UCUGUGAGACCAAAGAACUACUUU | UUGUUCUUUGGUCUUUCAGCC | 1 | chimp, mouse |
| miR-sb659 | AACCCUUUCCUAUAGUUGUUGCU | AACAAAUACAGGAAAGAGUUCU | 10 | chimp, macaque |
| [miR-sb105](file:///C:\Users\Dani\Desktop\DANAE%2029-01-13\Strong%20Lab\Strong%20Lab%20papers\miRNAs%20nuevos\expression\miR\sblock1051_novel.html)1 | ACGUGGAUUUGAAAUUAACCUC | UAUUAACAACAAAUUCCGG | 11 | - |
| [miR-sb121](file:///C:\Users\Dani\Desktop\DANAE%2029-01-13\Strong%20Lab\Strong%20Lab%20papers\miRNAs%20nuevos\expression\miR\sblock1217_novel.html)7 | CAUCUAUUUUUAUUGGUUCCU | AGUAUUUGUAAUAUAGGUUAAUG | 12 | - |
| [miR-sb205](file:///C:\Users\Dani\Desktop\DANAE%2029-01-13\Strong%20Lab\Strong%20Lab%20papers\miRNAs%20nuevos\expression\miR\sblock2058_novel.html)8 | CCCAGCCCCACGCGUCCCCAUGC | GGCGGGGCGUGUGCGGCUGCU | 17 | macaque |
| [miR-sb261](file:///C:\Users\Dani\Desktop\DANAE%2029-01-13\Strong%20Lab\Strong%20Lab%20papers\miRNAs%20nuevos\expression\miR\sblock2613_novel.html)3 | CCUGGUGCUCUGCCCCUCAGGA | CCUGUGGGGUGGAGCCCCUUGCUC | 2 | mouse |
| [miR-sb399](file:///C:\Users\Dani\Desktop\DANAE%2029-01-13\Strong%20Lab\Strong%20Lab%20papers\miRNAs%20nuevos\expression\miR\sblock3998_novel.html)8 | AAAUUAUUGUUAUUACUGAGUGUA | GUCUGUAGUGGCUAAGUUUC | 5 | chimp, macaque |
| [miR-sb436](file:///C:\Users\Dani\Desktop\DANAE%2029-01-13\Strong%20Lab\Strong%20Lab%20papers\miRNAs%20nuevos\expression\miR\sblock4360_novel.html)0 | GACACAUGACCAUAAAUGCUAAA | CUAGCAUUUAUAAUCAUGUGUUC | 6 | chicken, chimp, macaque |
