# Supplementary material for: Analysis of Novel NEFL mRNA Targeting microRNAs in Amyotrophic Lateral Sclerosis
Source: PLoS One. 2014 Jan 15;9(1):e85653. doi: 10.1371/journal.pone.0085653 (PMC3893244; doi:10.1371/journal.pone.0085653)
Supplement: Table S2 — Patient demographics of samples used in real-time PCR analyses. (DOCX) [file pone.0085653.s003.docx]

**Table S2.** Patient demographics of samples used in real-time PCR analyses.

| **Case** | **Gender** | **Age of death (yrs)** | **Site of onset** | **Duration (yrs)** |
| --- | --- | --- | --- | --- |
| Control | M | 74 | --- | --- |
| Control | M | 75 | --- | --- |
| Control | F | 68 | --- | --- |
| Control | M | 68 | --- | --- |
| Control | M | 67 | --- | --- |
| sALS | F | 75 | Bulbar | 1 |
| sALS | M | 72 | Limb | >5 |
| sALS | M | 78 | Bulbar | 8 |
| sALS | M | 71 | NA | NA |
| sALS | F | 71 | NA | NA |

---: not applicable

NA: not available
